# What I Know about Hay Fever

**Published:** 1905-10

**Authors:** E. Van Goidtsnoven

**Affiliations:** Atlanta, Ga.


### WHAT I KNOW ABOUT HAY FEVER.

By E. VAN GOIDTSNOVEN, M.D., Atlanta, Ga.

I have been a sufferer from hay fever for several years. Of its etiology I know but very little; of its treatment, still less. One thing I certainly know. It does not make a saint of the one who is afflicted by it. For further particulars I refer you to my friends Drs. Giddings, Willis Westmoreland and C. D. Smith. They know whereof I speak, and I apprehend it will be some time before their names and mine be presented for canonization. Henry Ward Beecher, who had hay fever, stated one day that it was an esthetic disease, affecting only those who had brains, blood, and money. Inasmuch as these three essentials seldom, if ever, harmonize or correlate so as to constitute such a complete and most desirable atavism, I am truly sorry I can not corroborate his statement as far as I am personally concerned. The most widely prevailing doctrine of the present day, respecting the origin of disease, is that of the "germ theory," yet it can not be controverted that there are many forms of disease which are, *per se*, *autogenetic* and derivative of certain states of lowered health, which are induced by visceral affections.

But what of those subtle forces incessantly at war, intangible, imponderable, imperceptible agencies, the one building up, the other destroying, not *material substances,* but rather *conditions of matter,* which man can neither create nor destroy, visible only in their manifestations, cataclysms, and survivals, perpetually existing, unchanging in quantity, yet ever changing in form? Compared to these, microbes and ptomaines play a very insignificant role as etiological factors.

Yet, I cheerfully and irresistibly subscribe to the germ theory and firmly believe hay fever to be a microbic disease. There arises every calendrical fall from the highlands of Africa a most violent current called sirocco. It is a hot, dry, dust-laden wind. During its prevalence persons suffer from great lassitude and exhaustion, and vegetation in Africa and contiguous coasts is parched and burned. This sirocco sweeps over seas and oceans. Its strength and violence are greatly modified before it reaches our shores. Nevertheless, the current is most keenly felt by neurasthenic people. To them it is most tangible. An inexpressible sense of malaise overwhelms them and migraine is one of the most common manifestations. Who has not heard of the baneful influence of east winds in autumn? I am strongly imbued with the conviction that this current is laden, not only with dust, but with a morbific agent, with a disease germ, either cryptogamic or microbic. I believe that this germ thrives only in certain zones, not unlike the isothermal lines, and that the atmosphere of some altitudes, like that of Roan Mountain, N. C., and others, destroy it. I am informed that the sway of this current stops at the Rocky Mountains, and that the Pacific coast is free from hay fever. I am rather partial to the microbic theory, because I do not think that cryptogams are as much affected by cold as the animated germ or microbe.

As I have stated above, neurasthenics are alone subject to hay fever. Hence the immunity of the great majority of mankind to this morbific agency. There must be a neurotic diathesis, a predisposition, or as biologists call it, a neurotic pabulum or soil before the microbe sets in and claims squatter sovereignty. The symptoms of hay fever are, in my humble opinion, but reflex actions due to irritation of Meckel's or spheno-palatine ganglion. Itching of the inner canthi, profuse lachrymation, profuse nasal secretion, frequent sneezing, itching of the palate and velum palate; of fauces; pharynx, posterior nares, and Eustachian tubes; and, finally dyspnea, are well-known concomitants of the disease. Meckel's ganglion supplies all these areas through the ascending branch which passes through the orbit, the descending branch through the palate, the internal branch through the nose, and posterior branches through the pharynx. I think the invasion takes place through the air-passages, and, in an afferent way, irritates the spheno-palatine ganglion; and the latter, in return, through its sympathetic plexus, affects the sensative areas above mentioned.

Neurasthenia, as a disease, can not be cured. The predisposition, therefore, remains the same. At each recurrence of the invasion the patient is inevitably attacked *de novo* and with renewed energy.

Hence, I do not think that there can be a cure for the disease. I do not believe that the removal of a deflected septum or portion thereof, or the obliteration of any other sensative area are curative proceedings.

The treatment, as applied by Drs. Brown and Campbell, of Atlanta, is eminently palliative, and has afforded me the greatest relief I have had in years. Through their skill and kindness I have this year been able to attend to my professional duties in a much more satisfactory manner than heretofore.

The microscope has proved to be the greatest bloodhound of the age. Pasteur, Koch, and San Domingo Freire are our modern heroes, and their names are indelibly engraved in the annals of medical lore and in the hearts of their fellowmen. But their unravelings have failed, so far, to verify the old saying that, "to point out the cause is to point out the cure."

To propose or to claim a cure for yellow fever, cholera, tuberculosis, smallpox, hay fever, etc., is a quixotic assumption. One might as well throw a glass of water into Vesuvius, or defy a cyclone with a popgun! There is no safety but in immunition, hygienic measures, and in most cases a masterly retreat.

Immunition is the remedy of the future. We are gradually, but surely and irresistibly, driven thereto by its preponderating and self-asserting claims.

Believing, as I do, that micro-organisms constitute the *fons et origo* of hay fever, I can not but feel impressed with the conviction that the day is not far distant when the bacillus of this disease will assert itself, and its attenuated culture will be used to immunize, *secundum Pastoris ordinem,* those unfortunate neurasthenics who, like myself, are subject to its renewed attacks and diabolical influenza.

Hay fever! influenza! What technical expressions in this new era of medicine! How scientific, graphic, descriptive and suggestive a name for a neurotic disease! It reminds me of what Ingersoll says of clergyman's sore throat: "I call it Parsonitis. It's something auctioneers never have."

Since I wrote and published the above statement, it has been discovered by Cannon, and corroborated by Klein, Pfeiffer, Kitasato and others, that hay fever is of microbic origin, having its seat in the brain, distinguishing characteristics, accompanied by an intense hyperemia of the pia mater at the base of the brain, distended arteries and the consistence of the brain and spinal cord increased thus in a great measure corroborating the above statement which I published more than ten years ago. The Meckel's ganglion seems to be principally affected. Hence the special and reflex involvement of all the areas supplied thereby, and which are cited above.

The ablation of any of these areas results in no good, as they but manifest neuroses and certainly no primary foci of disease. The spheno-palatine or Meckel's ganglion supplying, as it does, branches to the lining membranes of the nose, inner canthi, velum palati, pharynx, Eustachian tube and bronchi, etc., it is easy to understand the sympathy that exists between them and their interchangeable neuroses and the continuity of the membranes throughout these various organs must, in my opinion, satisfactorily account for all the symptoms produced in one part by impressions upon another. All these symptoms are examples of reflex nervous impulses and these intimate sympathetic relations between various portions of the animal economy exhibit themselves with exceptional forces in patients of nervous temperaments.

There are two most distressing concomitants of hay fever, one in the acute form, asthma; the other should be classed rather as an outcome that is neurasthenia or rather cerebrasthenia. This form of nervous exhaustion is not a disease, but a condition, the progressive and ultimate result from the microbic invasion. All the functions which maintain the integrity of the nerve cells have been overstrained, paralyzed as it were by shock or deranged by trauma, and, in the language of a famous neurologist, all osmosis is inhibited and cell function is disordered or deficient. Hence, as long as the laws of psychical, mental, cerebral and spinal are ignored there will be little or no response to any form of treatment, be it systemic or local. Neurasthenia means not only cell exhaustion, but cell starvation, for want of lecithen. On the face of this, according to the same authority, treatment would appear to be a simple matter, merely to suspend the cause and increase nutrition, but the nutritrive processes are not so ordinate with the increment of nutrients, and bounteous plenty about a starving man, too weak to digest, or to help himself, is of no avail. Appease the brain centres, guard against all emotional influences, except those of a pleasant and recreative character. Resort to psychical agences, improve the environments. Move your patient to a zone and altitude uncongenial to the growth and propagation of the germ, thereby effecting a masterly retreat against so many odds, and you secure as safe, warrantable and judicious a treatment as can be obtained from the list of all available means hitherto discovered.

Many, yea countless, are the remedies now in vogue and lauded for this dreadful disorder, especially those of local application, but, alas, they are loaded with dope, and it can not be controverted that many specialists are to-day responsible for the thousands of cocaine and morphine fiends that are now filling our sanitaria and state prisons. Dipsomania has also increased to a fearful extent, owing to the insatiable craving for a stimulant sufficiently potent to cope up with brain exhaustion.

That the name hay fever, or influenza, is a misnomer is self-evident to any one who has been afflicted with the disease for years. It designates a condition to which numerous other terms have been applied with a great fitness. This section of Georgia is no area and dust fever might be applied with equal fitness. To the array of names already ill-chosen, because they are misleading, I have the temerity and audacity to add another; and I propose to call it cerebral or *brain catarrh.* And so it is, and here I challenge the attention of all those who have witnessed or experienced the manifestations through the cribiform plate. Coriza is a nervous catarrh of a disordered brain. To the least attentive observer of health and disease the mucous membranes must always rank amongst the most interesting and important of the tissues which compose the human fabric. In an anatomical point of view their distribution betrays evidence of the most exquisite design, the greatest possible diversity of figure and arrangement being resorted to for the purpose of affording a prodigious extent of mucous surface. So perfectly is this object effected that the mind of even an anatomist would be absorbed with wonder could it, at a glance, behold spread out on a plain surface, the space these membranes really occupy, and the immense extent of their ramifications through cells, tubes, canals, reduplications and convolutions in almost every variety of arrangement and form. They compose, it may be said, the groundwork on which most of the vital functions of secretion, excretion, and absorption are effected; and besides this, are intimately concerned in the perfection of the sense of sight, hearing, smell, taste, and infinitely more than has been yet heretofore imagined, the faculty of speech. In the consideration and treatment of hay fever, our attention should be given to the division of the great mucous tract, ramifying throughout the respiratory and intestinal organs. We are all acquainted with the *modi operandi* now in vogue in the topical treatment of hay fever, and their names are legion.

I maintain that this disorder is essentially due to an abnormal susceptibility of nervous tissue, such as neurasthenia, neurasthenic heredity, functional disturbance of a brain centre, controlling the mucous membranes with their reflex neuroses, without necessarily destroying life, unless being unchecked by cell nutrients it inevitably ends in sheer vital exhaustion.

For many years I have consulted with my fellow sufferers as to the periodicity of the disease, and without a single exception I have obtained the information that the 26th of August is the grand opening day! It is hardly reasonable to assume that the pollen of various plants, which give rise to attacks in different individuals, will be set free to float away on their fructifying pilgrimages on exactly the same day and at nearly the same hour each recurring year and that they will reach the nostrils of sufferers, in their varying localities and situations and vocations, simultaneously, year after year. The nerve theory overshadows with equal force the pollen theory, for it shows that the disease exists under conditions which are the least favorable to the operations of the pollen.

### TREATMENT.

My personal experience, corroborated by a somewhat extended practice amongst fellow sufferers, has most forcibly impressed upon me the necessity of first restoring the nervous system to a healthy action, hence as a cell nutrient protonuclein associated with a cell sedative and calmative I know of none more adapted to the case than dionin and cannabis indica. Beware of morphine and cocaine in any form! Sulphate of atropia will control nasal lachrymal secretion, nausea and vomiting; podophyllin and caffeine will most effectively remove all hepatic and renal obstructions. The effects of the suprarenals or adrenals, although prompt and effective, are of short duration. I have found their combination with blennostasine a most useful one. Hence I prescribe as follows:

Dionin:
Cannabis Indica..............āā gr. 5
Caffeine.................. gr. 32
Adrenal or Suprarenal.............. gr. 32
Blennostasine.................. gr. 32
Pododophyllin.................. gr. $2\frac{1}{2}$
Protonuclein.................. gr. 64
Div. in caps. xxxii.

Take one capsule at 7, 11, 3 and 7 o'clock.

Blennostasine, a preparation put up by that most reliable house, McKesson & Robbins, is a substitute for quinine as a remedy for colds, superior to belladonna and its preparations for diminishing extensive mucus secretions. I employ it as a vasomotor constrictor for its drying or blennostatic effects in the treatment of hay fever, influenza and catarrhal hypersecretions. It is a nontoxic, and preferable to quinine, atropine, belladonna and similar drugs.

One of my patients calls the above "*sunshiners*" and I must say he has many fellow sponsors.

Personally, I use no other treatment than the above mentioned, and I now put all my patients under the same with the most satisfactory results. In the majority of cases it precludes all other forms of treatment. But, should the necessity arise for conjoined treatment, I would recommend the following:

A. As a spray.

℞. Quinine muriate..................gr. x
Atropia sulph..................gr. i
Aqua distillata..................f℥ i M.

This will stop all symptoms of coriza, but the effect will not be lasting unless followed by the following application:

B. Solution adrenalin chloride.

(Armours)..............................3.0

Menthol } ā ā.........................0.3

Camphor }

Lanolin..................................5.0

Mix and add olive oil q. s. ad 300 M.

Apply in nostrils shortly after the preceding.

C. Fothergill's treatment is the remedy *par excellence* for asthma or dypsnea, the most distressing of all symptoms. It is as follows:

℞. Tinct. lobeliæ..........................f℥ v

Ammonii iodidi...........................ʒ ii

Ammonii bromidi.........................ʒ iii

Syr. tolutani..............................f℥ iii M.

Sig. One (1) teaspoonful every one, two, three or four hours during the paroxysms. This gives relief in a few minutes, and sometimes the relief is permanent.

In conclusion, I will say that I know of no more distressing disorder than hay fever. Its acute duration is of three months. I must be pardoned when I say that they constitute three months of hell on earth. Ask any of the unfortunate victims and they will not hesitate to so denominate its horrors and terrors. Add to this six months of neurasthenic convalescence and you may form a faint idea of the true pitiable condition. Only three months grace!

In the treatment of this malady the physician should rely more on detailed attention and close sympathy than on medicine. For in the language of Lamartine, "Medicine is rather the disposition to than knowledge how to cure. The science of a physician is composed only of heart and guesses. The disposition to relieve is in itself able to do so in a degree. A physician should be a good man, and virtue is half of his genius."

